# Metabolic Scaling in Birds and Mammals: How Taxon Divergence Time, Phylogeny, and Metabolic Rate Affect the Relationship between Scaling Exponents and Intercepts

**DOI:** 10.3390/biology11071067

**Published:** 2022-07-18

**Authors:** Valery M. Gavrilov, Tatiana B. Golubeva, Giles Warrack, Andrey V. Bushuev

**Affiliations:** 1 Department of Vertebrate Zoology, M.V. Lomonosov Moscow State University, 119991 Moscow, Russia; tbgolubeva@list.ru (T.B.G.); a_bushuev@mail.ru (A.V.B.); 2 Zvenigorod Biological Station, M.V. Lomonosov Moscow State University, 119991 Moscow, Russia; 3 Department of Mathematics and Statistics, North Carolina Agricultural and Technical State University, Greensboro, NC 27411, USA; warrack.ag@gmail.com

**Keywords:** basal metabolic rate, time of divergence, endothermic animal, scaling, phylogeny

## Abstract

**Simple Summary:**

This study is based on a large dataset and re-evaluates data on the metabolic rate, providing new insights into the similarities and differences across different groups of birds and mammals. We compared six taxonomic groups of mammals and birds according to their energetic characteristics and the geological time of evolutionary origin. The overall metabolic rate of a taxonomic group increases with the geological time of evolutionary origin. The terrestrial mammals and flightless birds have almost equal metabolic levels. The higher the metabolic rate in a group, the less it increases within increasing body size in this group.

**Abstract:**

Analysis of metabolic scaling in currently living endothermic animal species allowed us to show how the relationship between body mass and the basal metabolic rate (BMR) has evolved in the history of endothermic vertebrates. We compared six taxonomic groups according to their energetic characteristics and the time of evolutionary divergence. We transformed the slope of the regression lines to the common value and analyzed three criteria for comparing BMR of different taxa regardless of body size. Correlation between average field metabolic rate (FMR) of the group and its average BMR was shown. We evaluated the efficiency of self-maintenance in ordinary life (defined BMR/FMR) in six main groups of endotherms. Our study has shown that metabolic scaling in the main groups of endothermic animals correlates with their evolutionary age: the younger the group, the higher the metabolic rate, but the rate increases more slowly with increasing body weight. We found negative linear relationship for scaling exponents and the allometric coefficient in five groups of endotherms: in units of mL O_2_/h per g, in relative units of allometric coefficients, and also in level or scaling elevation. Mammals that diverged from the main vertebrate stem earlier have a higher “*b*” exponent than later divergent birds. A new approach using three criteria for comparing BMR of different taxa regardless of body mass will be useful for many biological size-scaling relationships that follow the power function.

## 1. Introduction

Metabolic scaling, including the context-dependent influence of many modal effects, has been actively discussed for over 90 years [1,2,3,4,5,6,7,8,9,10,11,12,13,14,15,16,17,18,19,20,21,22,23], with obvious progress. A number of factors have been proposed to explain the observed variation in both the scaling intercept and slope [2,3,4,5,6,24,25,26,27,28,29,30,31,32]. Certain theoretical foundations have been summarized in the Metabolic Theory of Ecology (MTE) [8]. Important additional theoretical perspectives include the metabolic-level boundaries hypothesis and the contextual multimodal theory (CMT) to explain variation in both the scaling intercept (“*a*”) and slope (“*b*”) [3,4,24,32].

Scaling patterns differ taxonomically with respect to physiological or developmental state, ecological lifestyle, and environmental conditions [3,5,6,24,32]. Insufficient attention is given to how certain theoretical frameworks (e.g., the metabolic-level boundaries hypothesis) explain the diversity of metabolic scaling observed, including variation in both “*a*” and “*b*” and their covariance (as also shown by [2,3,5,11,31,32,33].

Here we wish to draw attention to evolutionary allometry, which reflects long-term effects of adaptation, genetic, and developmental constraints, and phylogenetic inertia on metabolic rates averaged by species as endotherm clades diverge in geological time [34,35,36,37,38,39]. Evolutionary metabolic allometry has been shown to provide a strong link between the ecology of metabolism and macroevolution [39,40,41,42,43,44,45,46,47,48,49,50,51].

Without going into details of the history of the study of the metabolic rate in birds and mammals, we note that from the first comparative studies of the basal metabolism in birds and mammals a power function was used:

BMR = *a* m*^b^* or log(BMR) = log(*a*) + *b* log(m),

where “*a*” is the antilogarithmic of the logarithmic intercept (scaling coefficient) and “*b*” is the scaling exponent (slope in the logarithmic plot).

The coefficient *b* is different for each class: in birds it is closer to 2/3, and in mammals to 3/4 [52,53,54,55]. Lasiewski and Dawson [56] showed that the basal metabolic rate of passerines is 40% higher than that of all other birds. When passerines and non-passerines were analyzed separately, these authors obtained almost the same values for both groups (0.723 and 0.722, respectively), both of which are already close to 3/4, but there were significant differences in the coefficient *a*. It has since been generally accepted that birds have an exponent of 3/4 or between 2/3 and 3/4 depending on the taxonomic/ecological composition of the sample [11,22,57,58,59]. The most recent reviews for mammals report a value of 0.735 [60,61]; for all birds it is 0.667 = 2/3 [59]. Therefore, it is currently unclear whether the difference in the scaling coefficient *b* and the allometric coefficient *a* in mammals and birds reflects methodology or biological reality.

We chose to compare scaling indices between infraclasses in mammals and birds. In mammals, we recognize three infraclasses: subclass Prototheria, and subclass Theria divided into two infraclasses of Metatheria (or Marsupialia) and Eutheria. Phylogeny and classification of birds is still debated [62,63,64,65,66]. All modern groups of birds belong to the infraclass Neornithes, or fan-tailed birds, which in turn is divided into two groups—Paleognathae, or ratite birds (this usually includes flightless birds such as ostriches, emus etc.), and Neognathae (this includes all other species). Neognathic birds form two clusters with respect to their basal metabolic rate: passerines, and non-passerines [59,67]. Therefore, we identified three major avian groups among the birds: Paleognathae, Neognathae-Non-Passeriformes, and Neognathae-Passeriformes, which should be analyzed separately.

Endothermic animals developed at least twice in the therapsid line—the mammalian line and the theropod–avian line. The development of the main existing groups of birds and mammals was not synchronous on the geological time scale. Birds and mammals formed endothermy on different morpho-physiological bases. Birds retained nuclei in their red blood cells, unlike mammals, who lost nuclei. In birds, the left aortic arch is reduced, but in mammals, the right arch is reduced. The venous system of birds is more similar to the venous system of reptiles than to mammals. In birds, the respiratory system is different to that of mammals. However, both birds and mammals were able to form the level of metabolic activity (BMR), which allowed them to lead a lifestyle in a wide range of conditions. BMR includes energy costs for the continuous functioning of physiological systems (primarily blood circulation, and respiration). BMR is the energy expenditure of lying still at rest, awake, in the overnight postabsorptive state.

In attempts to estimate the varying slope of the metabolic scaling relationship in evolution, it has been suggested that the slope is either static across evolutionary time or changes at the major taxonomic groups [39,68]. Previous studies have examined the question by dividing a database into predefined taxonomic units and using them as independent replicates for testing the universality of scaling parameters. However, evidence for specific and universal scaling coefficients has been mixed and remains contentious [2,69,70,71,72,73,74,75,76]. Here, we focus only on two factors (of the many noted in the works of Glazier [2,3,24,32]) that have an explanation for the observed variation in both the intercept and the slope. First, we account for the geological time when the group evolved suggesting that younger groups would have higher metabolic demands, as shown by Zotin and colleagues [25,26,27,28], also [29,68] Atanasov and Dimitrov [29], and Gavrilov et al. [68]. Second, we have linked BMR with the level of activity because BMR is a proxy for the minimum metabolic power, which is required for “idling” the machinery that provides for any activity [24,77,78]. We recently showed that classes of terrestrial vertebrates exhibit the evolution of metabolic scaling. Both the allometric coefficient “*a*” and the allometric exponent “*b*” change differently depending on the geological time of group formation [68]. We found that the allometric exponent *b* regularly decreases from ectotherms to endotherms, while *a* is growing. An almost linear dependence is formed: the younger the class, the lower the allometric exponent. Here we test this pattern further by comparing six clades of endothermic vertebrates. We analyze BMR scaling in three major groups of mammals and three major groups of birds, which have an almost identical taxonomic rank (excluding passerines) and whose divergence time is well-dated. Here we analyze the changes in both the allometric coefficient “*a*” and the allometric exponent “*b*” in six endotherm clades, depending on the geological time of origin of the group, and compare these data with similar measurements of field metabolic rate (FMR) [79]. It is noted that correlation between *a* and *b* in multiple intersecting scaling relationships may be spurious, even when the relationships do not intersect [3,5,6,24].

Our research had the following objectives: (1) Find a best criterion for comparing BMR levels for different taxa regardless of body size. (2) Determine how the BMR levels in different taxa are related to the time of their divergence from the main stem of vertebrates. (3) Determine if there is a correlation between the average FMR of the group and the average BMR group level. (4) Determine whether there is a correlation in a biologically meaningful way between the scaling exponents and the levels of metabolic rate.

## 2. Materials and Methods

### 2.1. BMR Dataset

To establish a BMR dataset we combined the available databases of BMR of birds and mammals. We conducted a survey of the available databases of the basal metabolic rate (BMR) of birds [68]. We selected only data collected from adult, post-absorptive, resting individuals within their thermoneutral zones, because these criteria are required for metabolic rate measurements to be basal [80]. The phylogenetic tree and data on BMR of mammals were obtained from Genoud et al. [61].

These data are available in the online Supplementary Materials.

### 2.2. Date of Time of Divergence of Taxa

The sequence of emergence of the extant groups of endotherms may be presented as follows: monotremes (271 million years ago—mya), marsupials (193 mya), eutherians (115 mya) [81,82]. The emergence of the eutherians occurred almost simultaneously with the Paleognath birds (110 mya), followed by all non-passerine Neognathes (90 mya), and finally passerine Neognathes (ca. 50 mya) [83].

### 2.3. Statistical Analysis

The body mass and BMR data were log_10_ transformed before analysis to account for allometric scales. All scaling exponents in allometric equations used in our study were based on ordinary least squares (OLS) regressions of the form log (BMR) = *a* + *b* log(m), unless specifically mentioned. The statistical analysis was conducted in R version 3.6.1 [84]. To test for differences in allometric coefficients of regressions in different major groups of endotherms, we used an ANCOVA with log (BMR) as the dependent variable and log(m) as the covariate. To test for differences in slopes of the two regression lines, we tested the model with the interaction term of log(m) and the grouping factor versus the model without interaction using the ANOVA function in R. The differences between observed slopes and the theoretical slope of 3/4 were tested with a Student’s *t*-test. To estimate standard errors of allometric coefficient *a* we used the ‘delta method’ function from the ‘car’ package in R [84]. The significance level in all analyses was set as *p* = 0.05.

### 2.4. Level of BMR and Dimensionless Ratio of BMR

First, we applied a test of the homogeneity of the slopes, which in our case tests the null hypothesis H0: *b*1 = *b*2 = *b*3 = *b*4 = *b*5 = *b*6. We used the multiple linear regression procedure ‘lm’ in R, which permits both numerical and categorical predictor variables [85].

We then found the average BMR for all groups. We used three methods: a common weighted average slope calculated for all groups in two different ways. One of them used the sample size as weights, while the other used the sum of the squared deviations of the predictor variable, log body mass, as weights. The standard statistical practice is that when aggregating estimates by averaging, weights should be used that are inversely proportional to the variance of the estimates [84]. Either way, using these weights, we matched the slopes for each group.

After finding the common slope “*b*”, we find new coefficients *a* by the method of least squares in accordance with the following system of equations for all selected groups:

$$\log(a)\cdot n+b\cdot \sum_{i=1}^{n}\log\left(x_{i}\right)=\sum_{i=1}^{n}\log\left(y_{i}\right)$$

$$\log(a)\cdot \sum_{i=1}^{n}\mathrm{lg}\left(x_{i}\right)+b\cdot \sum_{i=1}^{n}\left(\log\left(x_{i}\right)\right)²=\sum_{i=1}^{n}\log\left(x_{i}\right)\cdot \log\left(y_{i}\right)$$

If we scale the *a*_i_ by dividing each one by the *a* for passerines, so that we have *a*_pass_ = 1, (since that is the largest, all the other *a*_i_ will be less than one), we obtain a dimensionless ratio measurement for BMR.

Third, for correctly defining scaling relationships *a* and *b* Glazier [3,86] suggests that the response variable can be replaced with another measure of scaling elevation, L, which is log (Y/X) at the pivotal midpoint of a log-log scaling relationship. For metabolic scaling relationships, this measure of elevation (metabolic level) may correlate in a certain way to both *a* and *b*. We aim to find evidence for biologically significant correlations between the elevation and slope of scaling relationships using the definition of both *a* and *L*, as well as the BMR ratio.

### 2.5. Phylogenetic Analysis

The avian phylogeny was extracted from the birdtree.org database (http://www.birdtree.org, accessed on 19 March 2020) using the study by Hackett et al. [63] as the basis for phylogenetic reconstruction. The avian tree construction method was detailed in Bushuev et al. [87]. The phylogenetic tree and data on BMR of mammals was obtained from the study by Genoud et al. [61]. We used the phylogenetic generalized least squares model (PGLS) to take the phylogenetic signal into account in allometric analyses [88,89]. We used the ‘PGLS’ function from the ‘caper’ package (v. 1.0.1) for R [90]. Phylogenetic signal in mass-independent BMR was estimated with Pagel’s lambda (λ) [91] via a maximum likelihood (ML) approach using the same function. To test the differences in intercepts and slopes of phylogenetic regressions in different groups of endothermic animals, we determined the significance of the group term and its interaction in PGLS model using the same function. 

## 3. Results

### 3.1. Allometry of Metabolic Rate in Endotherms

Allometric equation for BMR of all 1817 endothermic species was

BMR = 5.549 m^0.676^, 95% CI’s: *a* 5.549 ± 0.022, *b* 0.676 ± 0.004,

where BMR is in mL O_2_/h and m is body mass in grams; R^2^ = 0.906.

**Figure 1:**
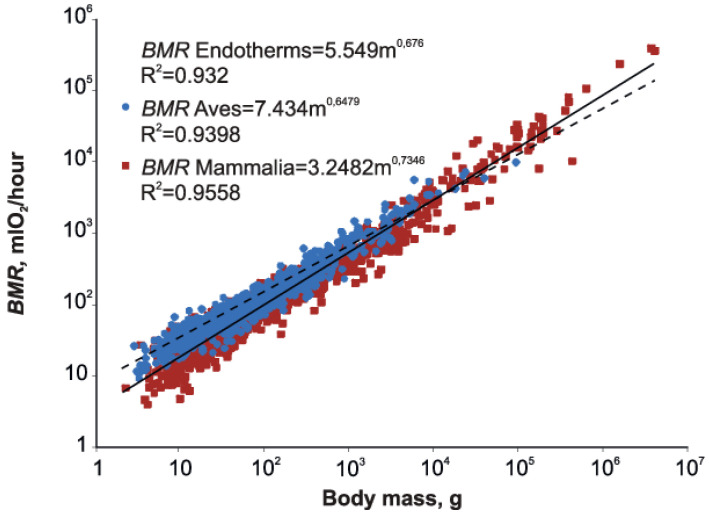
BMR as function of body mass in all endotherms and in mammals and birds separately. Solid line—mammals, dotted line—birds.

An examination of Figure 1 indicates that there is a noticeable difference between mammals and birds.

### 3.2. Mammalia vs. Aves

When the distinction between mammals and birds is introduced into the analysis (Figure 1),

Mammalia: BMR mL O_2_: avg mass = 20,064 g, avg BMR = 4703.20.

Aves: BMR mL O_2_: avg mass = 483 g, avg BMR = 407.72.

Mammalia: BMR = 3.248 m^0.735^ (*n* = 817, R^2^ = 0.956, SE(*b*) = 0.006).

Aves: BMR = 7.434 m^0.648^ (*n* = 1000, R^2^ = 0.940, SE(*b*) = 0.005).

The slopes (t = 11.558, DF = 1862) for Aves and Mammalia and the intercepts (*t* = −617.235, DF = 1862) for Aves and Mammalia are significantly different at *p* < 0.05. 

If we just have separate intercepts for birds and mammals and a common slope, we get a common slope of *b* = 0.7050 (SE = 0.0039).

Aves: BMR = 0.7725 m^0.7050^

Mammalia: BMR = 5803 m^0.7050^

The BMRs of birds are about 30–40% higher than mammals.

### 3.3. Allometry of Metabolic Rate in Major Clades of Mammals and Birds

**Figure 2:**
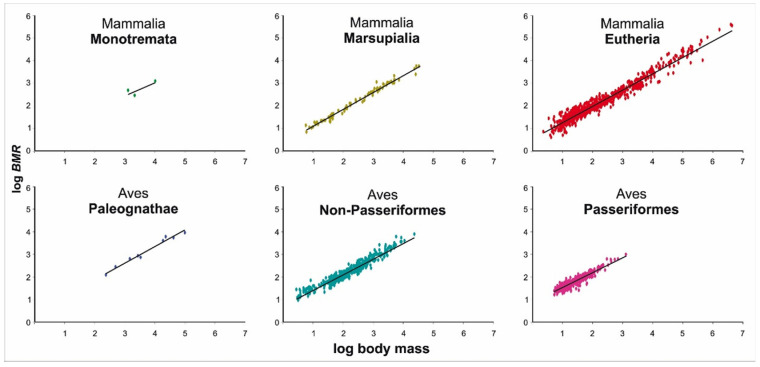
BMR as function of body mass in major groups of endotherms.

The scaling exponents of the major groups of endothermic animals ranged from 0.565 in Monotremata, 0.753 in Marsupialia, 0.736 in Eutheria, 0.727 in Paleognathae (flightless birds), 0.691 in Non-Passeriformes to 0.668 in Passeriformes (Figure 2, Table 1). The Monotremata dataset was characterized by a very narrow range of body weights (Table 1). The statistical significance of the dependence of metabolism on body size in this group arose from two data points for large species and is unlikely to be biologically meaningful. We exclude from further discussion the results of the allometric analysis of Monotremata regarding the slope of the regression line, as this group is represented in the database by only three species, but we will discuss their intercept.

The analysis of metabolic scaling in the studied taxonomic groups (Table 1) showed that the allometric equations for BMR differed in the allometric coefficient. Our results demonstrated that birds are clearly clustered into three significantly different groups. For the first time, we introduced a group of birds—flightless paleognaths (Paleognathae), in addition to the two previously described groups of Neognathes (non-passerines and passerines). Paleognaths differs from other birds in terms of energy characteristics. Importantly, the scaling exponents of the major groups of mammals and birds obtained by both the PGLS and the OLS analyses were almost identical (Table 1). Differences in allometric coefficients (a) were more pronounced (Table 1).

### 3.4. Allometry of Metabolic Rate in Major Clades of Mammals and Birds

We tested three types of regressions:

(1) Simple linear regression model: y = *a* + *b*x, y = log(BMR), x = log(m)
(2) Model with one slope and separate intercepts for each taxon: y = *a*_i_ + *b*x, i = 1,2,…,6
(3) Model with separate slopes and separate intercepts for each taxon: y = *a*_i_ + *b*_i_x

(If x = log (body weight) and y = log (BMR) we have an allometric model)

The index i varies according to the number of taxa groups. We computed R^2^ for all 3 models, also the Aikaike Information Criterion (AIC) and the Bayes Information Criterion (BIC) (see below). Here are the results:

(1) Simple linear regression
  Residual standard error: 0.1797 on 1815 degrees of freedom
  Multiple R^2^: 0.9317, Adjusted R^2^: 0.9317 AIC = −1076.75, BIC = −1060.24
(2) One slope, separate intercepts
  Residual standard error: 0.146 on 1810 degrees of freedom
  Multiple R^2^: 0.955, Adjusted R^2^: 0.955, AIC = −1822.26, BIC = −1778.22
(3) Separate slopes, separate intercepts
  Residual standard error: 0.145 on 1805 degrees of freedom
  Multiple R^2^: 0.956, Adjusted R^2^: 0.956, AIC = −1847.0, BIC = −1775.44

The results of this test make the common slope hypothesis seem quite plausible. Thus, we conclude that the model with one common slope (0.7248) and *a* separate intercept for each of the major groups is the best one, in the sense that it is the simplest model explaining the data. Two widely used criteria for obtaining a model that combines accuracy in prediction with parsimony in the number of predictors are the Akaike’s Information Criterion (AIC) and the Bayes Information Criterion (BIC). AIC and BIC should have scores that are as low as possible. Of the three models, Model 2 has the lowest BIC (−1778.22) and a low AIC = −1822.26.

Using another method to find the common slope for all groups, we calculated a weighted average of the slopes group using n_i_, the group sample sizes as weights. Using these weights, we matched the slopes for each group, PGLS (*b* = 0.698) and OLS (*b* = 0.704): PGLS: *b* = ((0.565 * 3) + (0.746 * 84) + (0.733 * 741) + (0.727 * 9) + (0.708 * 404) + (0.642 * 587))/1817 = 0.698; OLS: *b* = ((0.565 * 3) + (0.753 * 84) + (0.736 * 741) + (0.727 * 9) + (0.691 * 404) + (0.668 * 587))/1817 = 0.704.

We recalculated the original equations with a three commons average *b*, by using standard least-squares regression methods, for PGLS (*b* = 0.698), OLS (*b* = 0.704) and (*b* = 0.7258) and obtained new allometric coefficients (Table 2).

We now scale the coefficient *a* so that for passerines *a* = 1 and for the rest *a* becomes *a/a*_Pass_ to obtain a dimensionless ratio of BMR relative to the BMR for passerines (Table 2). This BMR ratio showed no significant differences in values obtained both with and without taking into account phylogeny (Table 3).

Thus, we obtained three versions of size-independent BMR in different clades of endotherms, which characterize the average value of BMR in the clade: *a*, mL O_2_/h at the common slope of 0.7248, relative passerine BMR (BMR ratio) and scaling elevation (metabolic level), L. This allows comparison of the BMR of a group depending on its evolutionary age, and further comparison of these data with similar measurements of field metabolism (FMR).

Combining the separate slopes for each group is not generally the best way to estimate a common slope. Therefore, in the following analysis, we use the common slope of 0.7248, since it has the highest R^2^, but similar conclusions are obtained when using the slopes for PGLS (*b* = 0.698) and OLS (*b* = 0.704).

For metabolic scaling relationships and comparison with the dimensionless ratio of BMR, we defined another measure of scaling elevation (metabolic level), L, which is log (Y/X) at the pivotal midpoint of a log–log scaling relationship: Monotremata—−0.796; Marsupialia—−0.201; Eutheria—−0.509; Paleognathae—0.301; Non-Passeriformes—−0.018; Passeriformes—0.420.

This metric gives similar results with the dimensionless ratio of BMR, with the highest level in passerines, and the lowest one in Monotremata.

**Figure 3:**
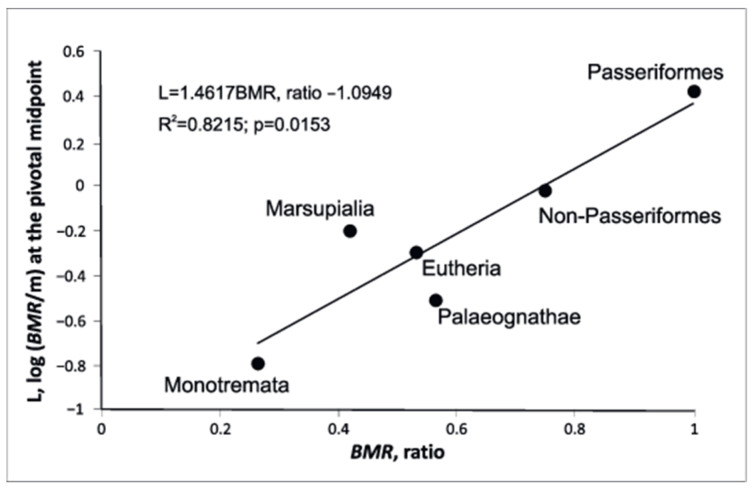
Relationship between scaling elevation, L and the dimensionless ratio of BMR.

Since in this paper we are using new indicators of metabolic rate for the first time, we compared the ratio between L and the dimensionless ratio of BMR (Figure 3). The analyses of the six groups illustrate that the variation in metabolic scaling relationships is systematically related to metabolic level.

### 3.5. Metabolic Allometry and Divergence Time of Various Groups of Endotherms

Allometry indices in groups of endothermic animals vary with their evolutionary age.

**Figure 4:**
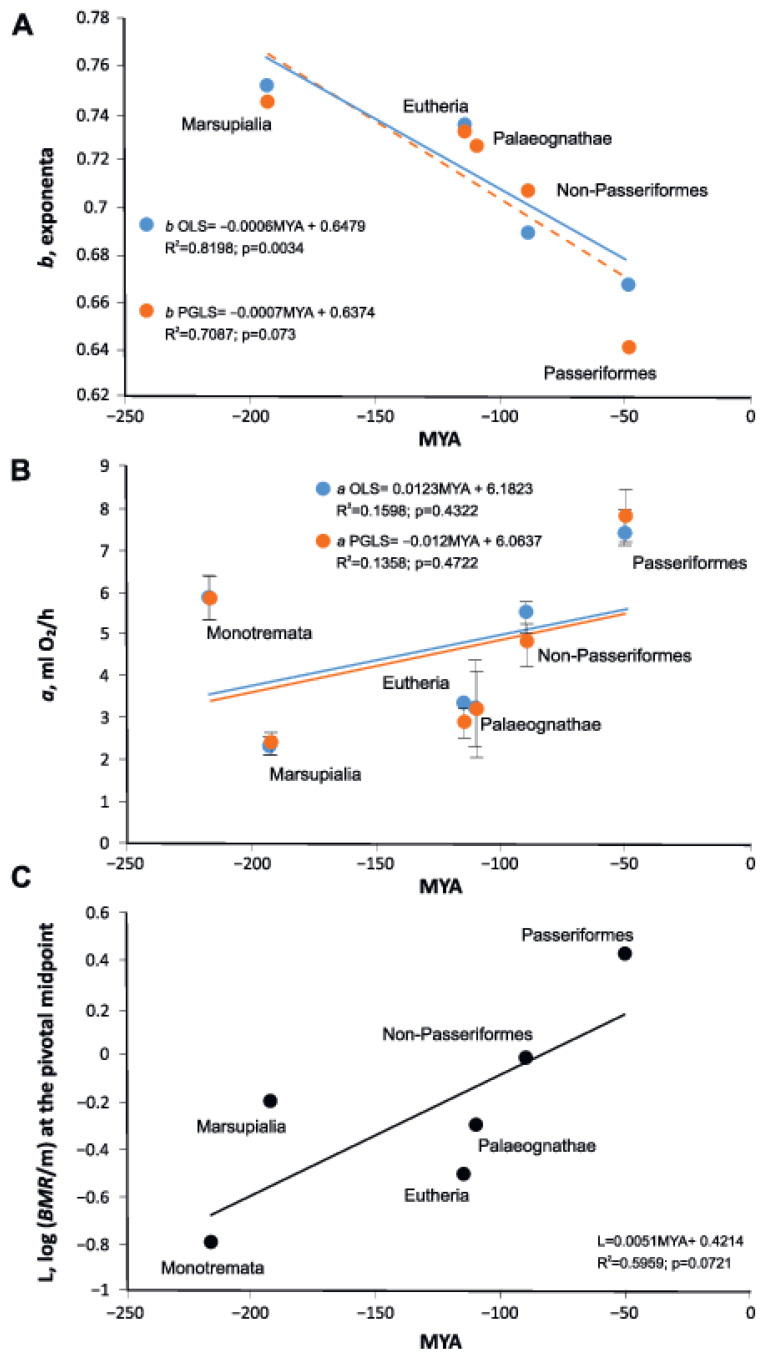
(**A**) Scaling exponents in five groups of endothermic animals’ dependence on the geologic time of divergence of the clades obtained by the phylogenetic generalized least squares (PGLS) and ordinary least squares (OLS). (**B**) The allometric coefficient in six groups of endothermic animals’ dependence on the geologic time of divergence of the clades by the phylogenetic generalized least squares (PGLS) and ordinary least squares (OLS). (**C**) The measure of scaling elevation, L, in six groups of endothermic animals’ dependence on the geologic time of divergence of the clades by ordinary least squares (OLS). MYA—million years ago.

The level of metabolism, as shown by the allometric coefficient and measure of scaling elevation, L, increases in younger groups, while slope *b* in the group decreases with the geological time of group formation (Figure 4). An almost linear dependence is observed: the later a group evolved, the lower its allometric exponent and the higher its allometric coefficient *a* and measure of scaling elevation, L (Figure 4).

The metabolic rate per 1 g, *a* from the original regressions of PGLS and OLS, depends on the divergence time of the group, but for OLS regression it is not significantly different from 0 at *p* = 0.05. However, if we use WLS regression, *p* < 0.05.

**Figure 5:**
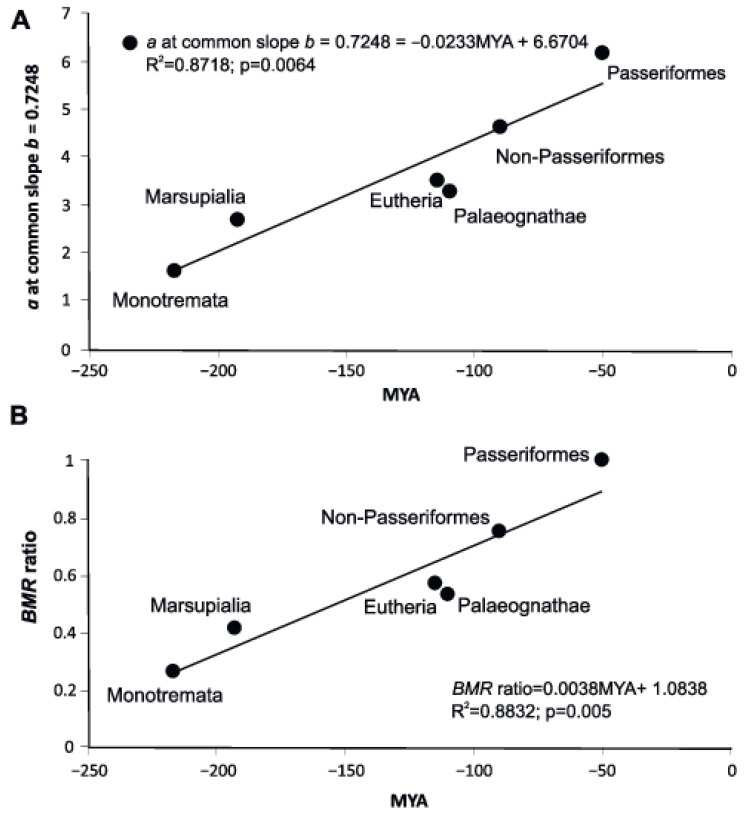
(**A**) The allometric coefficient *a* in different groups, after recalculation of the equations and transforming with a common average *b* = 0.7248 using the normal OLS procedure depending on the geologic time of divergence of the clades. (**B**) *BMR* level represented as BMR ratio in the six major groups of endothermic animals depending on the geological time of appearance of the group in evolution. MYA—million years ago.

Applying regressions with a common slope *b* = 0.7248 sharply increases both R^2^ and the reliability of the regressions, and to a higher degree when using the dimensionless BMR ratio (Figure 5).

The metabolic rate increases as the time of group divergence approaches the present, both represented as BMR ratio and as a measure of scaling elevation, L, which is log (BMR/m) at the pivotal midpoint of a log–log scaling relationship. BMR ratio regressions have significantly higher R^2^ and higher confidence levels.

### 3.6. FMR (Field Metabolic Rate), BMR, and Divergence Time of Various Groups of Endotherms

We recalculated the data available in the literature for birds and mammals in the following allometric dependences on the body mass [79]: for mammals, FMR = 10.04 m^0.734^, *n* = 79, *p* < 0.0001, R^2^ = 0.950; and for birds, FMR= 21.85 m^0.681^, *n* = 95, *p* < 0.0001, R^2^ = 0.938, where FMR is in mL O_2_ h^–1^ and m is body mass in g.

These equations indicate that the amount of energy the birds spend for their life supporting activities is twice as much as mammals. At the same time, the difference in the BMR level in birds and mammals does not exceed 40%. Increased energy expenditure in birds for life supporting activities is due to the longer duration of activity.

We did adjust for body size effects by using an analysis of covariance as we did for BMR. This statistical method allows for the comparison of *a* among different groups which is reasonable since *b* is fixed (Table 4)

**Figure 6:**
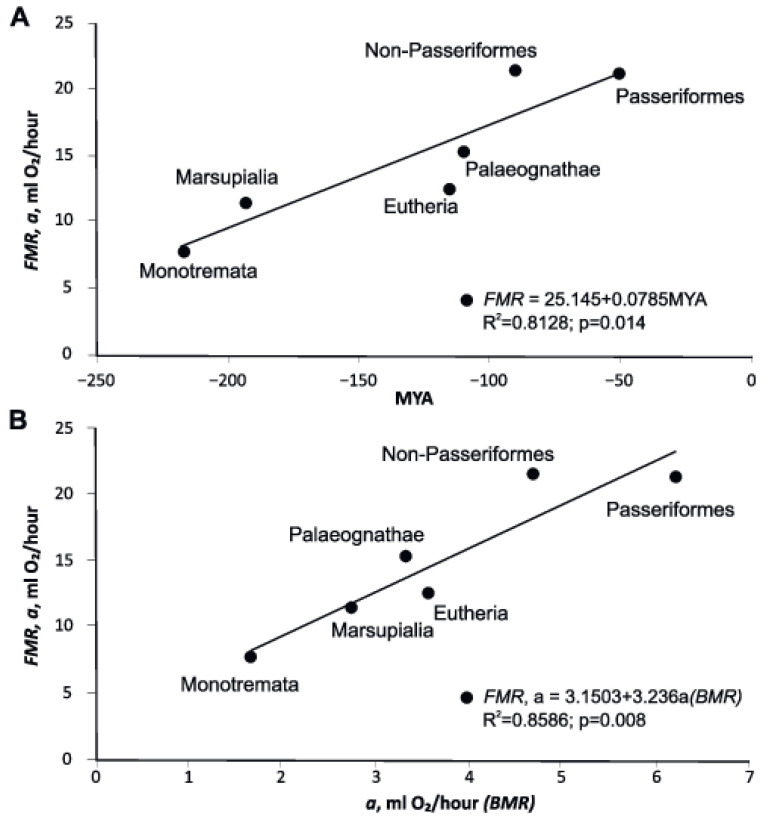
(**A**) The average FMR in the six major groups of endothermic animals depending on the geological time of appearance of the group in evolution. Regression lines and statistics in the figure are calculated using OLS method. (**B**) Relationship between FMR and BMR in the six major groups of endothermic animals. MYA—million years ago.

The BMR/FMR- ratio shows the proportion of self-maintenance costs out of total energy costs required for living in nature (Table 4, α = BMR/FMR). The BMR/FMR ratio is the lowest in Monotremata (0.209). The following are the values for other groups: Marsupialia—0.234, Eutheria—0.282, Paleognathae—0.215, Non-Passeriformes—0.216, and Passeriformes—0.290. Flightless Paleognathae and Eutheria have almost identical relative BMR, but the BMR/FMR ratio in Paleognathae is significantly lower. Size-corrected FMR increases in evolutionarily younger groups (Figure 6, Table 4).

The mass-corrected FMR in the six groups of endothermic animals is positively associated with the mass-corrected BMR, (Figure 6). 

In the databases for BMR and FMR, there are species for which both BMR and FMR are measured. We identified these species and calculated the ratio of BMR to FMR for them. Avian species that already have a higher BMR increase energy expenditure at FMR more significantly than mammals (2.75 in mammals vs. 3.49 in birds, the averages are different at *p* = 0.05, t = −11.05, df = 110). That is, the usual vital activity of birds is provided by almost two-fold energy expenditure in comparison with mammals. Note that the mass exponents in birds and mammals in the dependences of FMR on body weight do not significantly differ from slopes in BMR.

### 3.7. Relation between the Scaling Exponents and the Allometric Coefficients of Evolutionary Groups

We have established how the exponent “*b*” depends on “*a*”, the allometric coefficient of metabolic rate per gram of body weight in five groups of endotherms (excluding Monotremata). We obtained the following linear dependences “*b*” on “*a*”, both being in units of mL O_2_/h per g of body weight:

*b*_OLS_ = 0.79 − 0.0185*a*, R² = 0.987, *b*_PGLS_ = 0.788 − 0.017*a*, R^2^ = 0.981.

**Figure 7:**
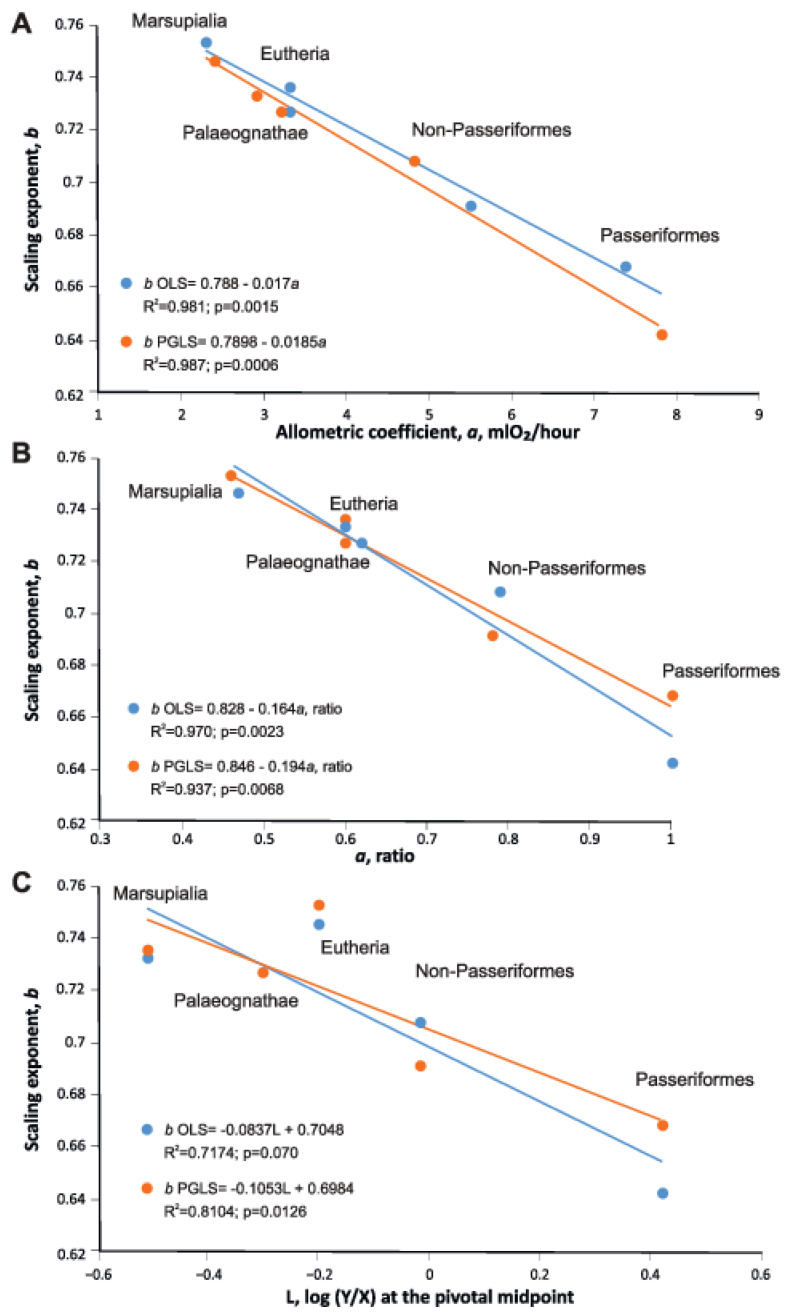
Dependence of scaling exponents on the allometric coefficients “*a*” (the level of metabolism) in five groups of endotherms: (**A**) “*a*” in mL O_2_/h per g. (**B**) “*a*” (ratio). The scaling exponents and the allometric coefficients were obtained by the ordinary least squares model (OLS—blue line) and phylogenetic generalized least squares model (PGLS—yellow line). (**C**) Dependences of the scaling exponents on the level of metabolism a measure of scaling elevation, L, which is log (BMR/m) at the pivotal midpoint of a log–log scaling relationship) in five groups of endotherms.

In relative units of “*a*”: 

*b*_OLS_ = 0.828 − 0.164*a* ratio, R² = 0.970, and *b*_PGLS_ 0.846 − 0.194*a* ratio, R2 = 0.937 (Figure 7). 

Linear relationships between the scaling exponents and the level of metabolic rate expressed both in mL O_2_/h per g and in relative units, when OLS and PGLS were used, show that the higher the standard metabolic rate in the group, the slower the metabolism grows with the increase of body mass in this group (R^2^ = 0.97)

Linear relationships between the scaling exponents and the level of metabolic rate expressed both in mL O_2_/h per g and in relative units (BMR ratio and measure of scaling elevation, L), when OLS and PGLS were used, show that the higher the standard metabolic rate in the group, the slower the metabolism grows with the increase of the body mass in this group (R^2^ = 0.97 and R^2^ = 0.81 at L).

## 4. Discussion

We have characterized the long-term evolutionary dynamics of the metabolic scaling relationships, in relation to the development of endothermic animals and endothermy proper. Despite the considerable history of discussion of metabolic scaling, as well as the substantial number of empirical tests, the available data show that there is not one scaling pattern, but a diversity of patterns.

Different groups, data sets, standardizations, and analytical approaches have provided different answers [1,2,3,4,5,7,8,10,12,13,14,15,16,17,18,19,20,21,22]. The association of scaling parameters with evolutionary age has been studied by Zotin et al. [24,28], Atanasov and Dimitrov [29], Ueda et al. [39], and Gavrilov et al. [68] on animals of different classes. In this report, we have applied three different methods of determining the metabolic rate and two methods of determining the slope in six groups of endothermic animals only. We paid special attention to the relationship of *a* and *b* in evolution. We consider transitions in metabolic scaling through the main groups of endotherms that diverged from the main trunks of birds and mammals at different times.

It is well known that the transition to endothermy causes a major shift in the value of the intercept of the allometric relationship [3,4,39]. Discussing the functional meaning of the allometric coefficient *a* in different groups of animals is very difficult due to its strong dependence on the scaling exponent, which varies greatly between taxa, of both different and similar taxonomic rank. We aimed to develop a method to compare BMR across groups, regardless of body size. We believe that the dimensionless ratio of BMR in different clades of endothermic animals will be a great help in comparative studies. We ran a test which showed that the model with one common slope and separate intercepts for each of six of the major groups of endotherms best fits the dataset. Thus, we obtained three versions, independent of both the size of the animal and of the BMR in different clades of endotherms, which characterize the average value of BMR in the clade: *a*, mL O_2_/h at the common slope, relative passerine BMR (BMR ratio), and scaling elevation (metabolic level), L for all six endotherm clades and found a correlation with the time of their divergence from the main trunks of birds and mammals. Our estimate of the dimensionless coefficient for six endothermic clades in a first approximation is consistent with the data obtained by McNab [58,59] both for birds and for mammals. We have characterized the long-term evolutionary dynamics of the metabolic scaling relationships, in relation to the development of endothermic animals and endothermy proper.

The analysis of metabolic scaling in the studied taxonomic groups (Table 1) showed that birds are clearly clustered into three significantly different groups. For the first time, we introduced a group of birds—flightless paleognaths (Paleognathae), in addition to the two previously described groups of Neognathes (non-passerines and passerines), and paleognaths differ from other birds in terms of energy characteristics. Importantly, the dimensionless BMR ratios for all groups of mammals and birds obtained by both the PGLS and the OLS analyses were identical (Table 2).

Variation in the scaling exponent (slope) was caused primarily by the fact that mammal and bird datasets included species from the six major groups in various proportions. Different slopes appear due to different BMR values across taxonomic groups, sample sizes, range of body mass across taxa, and different representations of species within each group. For example, the overall slope for all birds, *b* = 0.674, is obtained due to the highest BMR of passerines, which are concentrated in the lower part of the size range, and the lowest BMR of paleognaths, which form the upper part of the size range.

We assumed *a priori* that the slope is an evolutionarily labile trait. Atanasov and Dimitrov [29] demonstrated for taxa of different rank that the power coefficient *b* decreases along with the evolution of animals. In studies on shifts in metabolic scaling across major evolutionary transitions, prokaryotes, protists, and metazoans display a change in scaling from *b* > 1 for prokaryotes, *b* ≈ 1, for protists to *b* < 1 in metazoans [36]. The metabolic-level boundaries hypothesis that explains diverse metabolic scaling in animals and plants [3] predicts that the scaling slope should vary mostly between 2/3 and 1 and that it should be related to metabolic activity. Uyeda et al. [39] showed shifts in metabolic scaling across the vertebrate phylogeny. An analysis of these and other works allows us to put forward a hypothesis that in the main groups of vertebrate animals, the exponent *b* will decrease depending on the time of divergence. This is the same as what we found when looking at the slopes in groups of endotherms. In the development of endothermic animals and endothermy, proper the differences in the intercept are more pronounced. We find five values of intercepts (the intercepts of Eutheria and Paleognathae are almost equal).

Our results raise a number of important questions, which remain open.

For example, how should we properly count the number of slopes? Much of the current debate concerns the value of b, namely whether it is equal to approximately 2/3 (Rubner”s law) or to 3/4 (Kleiber’s law). We have found that in the history of endothermic vertebrates, two well-documented shifts to new evolutionary regimes occurred with satisfactory theoretical explanations by the resource transport network models of West and colleagues which predicts *b* = 3/4 for all mammals and flightless birds and *b* = 2/3 for flying birds [16,92]. Both slopes are predicted by Glazier’s metabolic-level boundary hypothesis.

We analyzed how the parameters *a* and *b* in equations relating BMR to body weight in different groups of endotherms change with the time of occurrence of fossil evidence (Figure 4). We applied three measures of metabolic rate to show that observed correlations between “*a*” and “*b*” have biological meaning.

Comprehensive studies based on the various models corrected the final effect of the size: exponent *b* = 3/4 is suitable only within the limits of exceptionally large body masses and differences in temperatures. In these studies, a quadratic (non-linear) approximation was obtained for the relationship between BMR and body size [9,22].

We reviewed these body size complications in a previous publication [68]. To check how the range of weights in the sample affects the regression exponent, we restricted the samples of mammals and birds to those with a body weight of 10 kg or less. In this case, the sample of birds decreased by only six species (less than 1%), and the sample of mammals decreased by 73 species (approximately 9%). We obtained the following [68]: allometric exponent — mammals 10 kg or less, *b* = 0.703 compared with *b* = 0.735 for all mammals; birds 10 kg or less, *b* = 0.646 compared with *b* = 0.648 for all birds. While the scaling exponent remained virtually unchanged among birds, there was a significant decrease in *b* for mammals.

Kolokotrones et al. [22] noted the curvilinearity of metabolic scaling in mammals (concave upward, primarily in eutherians), which complicates comparisons of *b* between eutherians and birds. They showed that small eutherians have a similarly low *b* value to that of small flying birds, whereas large eutherians have a higher *b* value similar to that of relatively large flightless birds. This pattern suggests that variation in *b* is not simply related to the time of evolutionary origin. In the present work, we arbitrarily reduced the samples of birds (without Paleognathes, *n* = 925) and mammals (only placentals, *n* = 552) to a body weight of 1 kg. Using standard regression methods, we fit a model using separate slopes and separate intercepts for each of the two groups (passerines/non-passerines and Eutheria). For this model we obtained R^2^ = 0.8794. The model with separate slopes is fitted because the *p*-value for testing the hypothesis that the slopes are equal (*b*_Aves_ = *b*_Mammalia_) is *p* = 9.49 × 10^−10^. Since the *p*-value is so small, the hypothesis of equal slopes should be rejected. These are the equations of the model (in allometric form):

passerines and non-passerines less than 1 kg: BMR = 8.2159 m^0.6176^

Eutheria less than to 1 kg: BMR = 3.9062 m^0.6975^.

Both the slopes and intercepts of these regressions were significantly different from each other (*p* < 0.001).

A successive decrease in the size range of weights in classes leads to a decrease in *b*, both in mammals and birds. The difference in slopes is 0.087 between the entire data set, and 0.079 when the size range is less than 1 kg. 

Endothermy has formed in birds and mammals independently and in different geological ages. However, in both groups, endothermy originated as an effect of selection for improved aerobic metabolism which provided a higher level of activity. The physiological basis by which aerobic metabolism is able to maintain a high level of activity in birds and mammals is different: mammals and birds have a different division of venous and arterial networks, erythrocytes with or without cell nucleus, and different lung designs. Mammals evolved earlier than birds, and from less advanced amniotes. The physiological basis creating BMR in birds provides a higher metabolic rate, but this increases more slowly with increasing body weight than it does in mammals. Our results show that both of these values can be found in endotherms: *b* ≈ 3/4 for all groups of mammals and flightless birds and *b* ≈ 2/3 for flying non-passerines and passerines: 

BMR (All Mammalia + Paleognathes) (mL O_2_/h) = 3.252 m^0.734^ (*n* = 826, R^2^ = 0.957, SE(*a*) = 0.033, SE(*b*) = 0.005) vs. BMR (Non-Passeriforms + Passeriformes) (mL O_2_/h) = 7.402 m^0^.^649^ (*n* = 991 R^2^ = 0.936, SE(*a*) = 0.023, SE(*b*) = 0.005). The statistics for testing for equality of slopes (*t* = 11.066, d.f. = 1813) and equality of intercepts (t = −609.059, d.f. = 1813) are both highly significant (*p* << 0.001) so in each case the hypothesis of equality should be rejected.

These data suggest that the allometric relationship of metabolic rate with body mass is not governed by a single overarching design principle that applies to all vertebrates or to all endotherms, but instead depends on various constraints at different body sizes and levels of structural and functional organization. Apparently, the increased whole-organism metabolic rate that accompanies the transitions occurs at the expense of decreased efficiency of conversion of metabolic energy into biomass, as was postulated for prokaryotes, protists, and metazoans [36]. The mechanisms underlying this decrease in efficiency with increasing body size and complexity across the transitions are unclear. It is assumed that larger, more complex organisms must allocate relatively more metabolic energy for the acquisition and processing of food resources and relatively less for biomass production. Models of resource distribution through vascular networks suggest a decrease of metabolic rate as body size increases [16,93]. 

Our results also support the general argument of Kolokotrones et al. [22], that those metabolic constraints may play an important role in the evolution of body size, particularly towards the upper limits of the size range.

If we consider all classes of vertebrates, then the ectothermic classes have a slope *b* > 3/4 (Pisces, Amphibia, and Reptiles, [93]. Whereas the slope 2/3 has a clear physical explanation of the surface-to-volume ratio (Fourier’s law), fundamental models based on novel theories have been suggested to explain the slope 3/4 [12,13,16,17,18,19,94,95,96] etc. Numerous explanations based on physical constraints and various conditional biological and environmental factors, as summarized by Glazier [4], describe four major modal mechanisms for metabolic scaling including surface area (SA), resource transport (RT), system composition (SC), and resource demand (RD) related mechanisms, whose expression is modulated by various internal and external influences [97,98,99,100]. Our results for six groups of endotherms demonstrates that the scaling of BMR is implicit in the design of the body (and its systems) of endothermic animals. The system(s) can vary with a change in body weight, in relation to as m^3/4^ or m^2/3^. BMR is essentially the energy required to keep the molecular machinery of life operating at zero activity. The coefficient *a* displays the level of development of these systems: the higher it is, the more developed the system is. Flight demanded an intensified development of these systems, which led to an increase in *a* in non-passerines and, even more so in passerines. 

An increase in *a* led to a decrease in *b*. This is consistent with the theoretical Glazier model’s assumption that a decrease in the slopes of the regression lines correlates with an increase in the BMR level (in particular, model of MLBH [3,4,24,32]). It should be emphasized that during transitions to a new higher regime of energy expenditure—field metabolism in natural conditions, or the energy of existence in captivity, no decrease in the slopes of the regression lines is observed (see section FMR, BMR, and divergence time of various groups of endotherms in the present article, and also Gould [30], Sieg et al. [74], and McClain et al. [75]). A decrease in the slope is present only in the evolutionary increase in the metabolic rate, as we have shown for five clades of endotherms, tetrapods [68], and in a broader aspect for prokaryotes, protists, and metazoans [36]. In the latter case, *b* decreases, and an increase in the level of metabolism may not occur (prokaryote yields calculated metabolic rates higher—not lower—than those of protists and metazoans (see Figure 1 in [36]). It has been shown that animals are organized in such a way that the specific metabolic power of important functional systems is maintained near the optimum, which does not depend on body size [101]. Furthermore, various principles of the spatial distribution of the metabolically active biomass inside the organism lead to various allometric dependences [102,103]. The reason behind this is that activity and muscle mass are both closely related to the total volume of mitochondria and capillaries of muscles [38]. The dependence of SMR (standard metabolic rate) on body weight in ectotherms tends to have an exponent >3/4 (0.78–0.88) [31,92,104]. Mammals that diverged from the main vertebrate stem earlier have a higher “*b*” exponent than later divergent birds. At the ecosystem level, mass-specific energy consumption declines with increasing animal body size in stable ecosystems [35,105,106]. At the same time, decreasing mass-specific energy consumption in large animals entails a decrease in the efficiency of the transition of metabolic to mechanical capacity and leads to a decrease in the mass exponent for BMR.

## 5. Conclusions

Although we have only metabolic data for extant species, we show that the relationship between mass and metabolic scaling has been changing throughout evolutionary history and has evolved across vertebrate lineages, and we indicate a historical trend in the development of metabolic scaling. In summary, we emphasize that this study draws on a large dataset of mammals and birds, demonstrating key trends in metabolic rates between endotherm groups. The dimensionless ratio of BMR in different groups of endotherms increases from Monotremata through Marsupialia to Eutheria in Mammalia, and from Paleognathae through Non-Passeriformes to Passeriformes in birds. If the highest BMR of Passeriformes is taken to be 1.00, the relative BMR level of Monotremata will be 0.264, Marsupialia 0.435, Paleognathae 0.532, Eutheria 0.571, and Neognathae-Non-Passeriformes 0.752. The terrestrial lifestyle of Eutheria and flightless Paleognathae is ensured by an almost equal level of BMR. Importantly, this study showed that the increase of metabolic levels in principle groups of endothermic animals negatively correlates with their evolutionary age: the later the group separated from the main the trunk of vertebrates, the higher is the metabolic rate. We found negative correlations between the scaling exponents and the allometric coefficients *a*, in five groups of endotherms and obtained linear dependences between the scaling exponents and the allometric coefficients *a*, both in units of mL O_2_/h per g (R² = 0.9769), and in relative units of allometric coefficients a (R² = 0.9718) and level or scaling elevation, “L” (R² = 0.8104). A decrease in the slope is present only within the evolutionary increase in the basal metabolic levels. During transitions to a new higher regime of energy expenditure FMR (field metabolic rate) decreases in the slope are not observed. Mammals that diverged from the main vertebrate stem earlier have a higher “b” exponent than later divergent birds.

## Figures and Tables

**Table 1:** Parameters of allometric equation for basal metabolic rate in main groups of endothermic animals.

Group	Number of Species	Body Mass Range, g	OLS: *a* ± SE	OLS: *b* ± SE	OLS: R^2^	Pagel’s λ	PGLS: *a* ± SE	PGLS: *b* ± SE	PGLS: R^2^
Mammalia	817	2.2–4,037,500	3.248 ± 0.107	0.735 ± 0.006	0.956	0.870	2.357 ± 0.632	0.735 ± 0.009	0.888
Monotremata	3	1284–10,300	5.861± 0.512	0.565 ± 0.387	0.681	0.000	5.861 ± NA	0.565 ± 0.387	0.681
Marsupialia	84	5.4–32,490	2.300 ± 0.152	0.753 ± 0.011	0.983	0.214	2.407 ± 0.222	0.746 ± 0.013	0.976
Eutheria	730	2.2–4,037,500	3.326 ± 0.115	0.736 ± 0.006	0.956	0.813	2.910 ± 0.393	0.733 ± 0.011	0.874
Aves	1000	2.8–92,400	7.435 ± 0.167	0.648 ± 0.005	0.940	0.664	5.514 ± 0.605	0.679 ± 0.010	0.830
Paleognathae	9	220.8–92,400	3.221 ± 1.147	0.727 ± 0.041	0.978	0.000	3.221 ± 0.871	0.727 ± 0.041	0.978
Non-Passeriformes	404	3.2–23,370	5.507 ± 0.262	0.691 ± 0.009	0.939	0.630	4.833 ± 0.589	0.708 ± 0.014	0.865

*Notes*. Allometric equation: BMR = *a*m*^b^*, where BMR is basal metabolic rate in mL O_2_/hour, m—body mass in g, *a*—allometric coefficient, *b*—scaling exponent, obtained from OLS and PGLS analyses.

**Table 2:** New intercepts for at different common slope.

Group	Number of Species	PGLS: *a,* mL O_2_/h at *b* = 0.698	R^2^ for *a* at *b* = 0.698	OLS: *a*, mL O_2_/h at *b* = 0.704	R^2^ for Regression at *b* = 0.704	OLS:*a*, mL O_2_/h at *b* = 0.7248	R^2^ for Regression at *b* = 0.7248
Monotremata	3	2.02	0.6570	1.92	0.6590	1.63	0.666
Marsupialia	84	3.14	0.9740	3.03	0.9760	2.69	0.980
Eutheria	730	4.07	0.9460	3.94	0.9480	3.53	0.952
Paleognathae	9	4.14	0.9740	3.93	0.9750	3.29	0.978
Non-Passeriformes	404	5.32	0.9360	5.16	0.9370	4.65	0.939
Passeriformes	587	6.72	0.8620	6.59	0.8640	6.18	0.868

**Table 3:** The dimensionless quantity of the BMR ratio in the main clades of endotherms defined in different ways.

Group	Number of Species	PGLS: *a*/*a*_Pass_ (BMR ratio)	R^2^ *b* = 0.698337	OLS: *a*/*a*_Pass_ (BMR ratio)	R^2^ *b* = 0.70449	Indicator Variables *a/a*_Pass_ (BMR ratio)	R^2^ *b* = 0.7248
Monotremata	3	0.3000	0.6570	0.2915	0.6590	0.264	0.666
Marsupialia	84	0.4670	0.9740	0.4600	0.9760	0.435	0.980
Eutheria	730	0.6054	0.9460	0.5977	0.9480	0.571	0.952
Paleognathae	9	0.6153	0.9740	0.5960	0.9750	0.532	0.978
Non-Passeriformes	404	0.7924	0.9360	0.7833	0.9370	0.752	0.939
Passeriformes	578	1.0000	0.8620	1.0000	0.8640	1.0000	0.868

**Table 4:** Relation between BMR and FMR in major groups of endothermic animals.

Group	BMR *a* at *b* = 0.7248	FMR *a* at *b* = 0.6851	BMR/FMR
Monotremata	1.63	7.81	0.21
Marsupialia	2.69	11.48	0.23
Eutheria	3.53	12.52	0.28
Paleognathae	3.29	15.33	0.21
Non-Passeriformes	4.65	21.54	0.22
Passeriformes	6.18	21.32	0.29

## Data Availability

The data presented in this study are openly available in in the online Supplementary Materials.

## Supplementary Materials

The following supporting information can be downloaded at: https://www.mdpi.com/article/10.3390/biology11071067/s1, Table S1: Mammalian BMR; Table S2: Avian BMR.
